# Liver Tumor Prediction using Attention-Guided Convolutional Neural Networks and Genomic Feature Analysis

**DOI:** 10.1016/j.mex.2025.103276

**Published:** 2025-03-22

**Authors:** S. Edwin Raja, J. Sutha, P. Elamparithi, K. Jaya Deepthi, S.D. Lalitha

**Affiliations:** a Department of Computer Science and Engineering, Vel Tech Rangarajan Dr. Sagunthala R&D Institute of Science and Technology, Chennai, India; b Department of Computer Science and Engineering, SRM Institute of Science and Technology, Ramapuram, Chennai, India; c Department of Artificial Intelligence and Data Science, Ramco Institute of Technology, Rajapalayam, Virudhunagar, India; d Department of Artificial Intelligence and Machine Learning, School of Computing, Mohan Babu University, Tirupati, Andhra Pradesh, India; e Department of Computer Science and Engineering, R.M.K. Engineering College, Kavaraipettai, Thiruvallur, India

**Keywords:** Liver tumor prediction, Multi-modal data fusion, Tumor segmentation, Tumor subtype classification, Attention mechanism, Medical Imaging, Radiomics, Attention-Guided Convolutional Neural Networks (AG-CNNs), Genomic Feature Analysis Module (GFAM)

## Abstract

The task of predicting liver tumors is critical as part of medical image analysis and genomics area since diagnosis and prognosis are important in making correct medical decisions. Silent characteristics of liver tumors and interactions between genomic and imaging features are also the main sources of challenges toward reliable predictions. To overcome these hurdles, this study presents two integrated approaches namely, – Attention-Guided Convolutional Neural Networks (AG-CNNs), and Genomic Feature Analysis Module (GFAM). Spatial and channel attention mechanisms in AG-CNN enable accurate tumor segmentation from CT images while providing detailed morphological profiling. Evaluation with three control databases TCIA, LiTS, and CRLM shows that our model produces more accurate output than relevant literature with an accuracy of 94.5%, a Dice Similarity Coefficient of 91.9%, and an F1-Score of 96.2% for the Dataset 3. More considerably, the proposed methods outperform all the other methods in different datasets in terms of recall, precision, and Specificity by up to 10 percent than all other methods including CELM, CAGS, DM-ML, and so on.

• Utilization of Attention-Guided Convolutional Neural Networks (AG-CNN) enhances tumor region focus and segmentation accuracy.
• Integration of Genomic Feature Analysis (GFAM) identifies molecular markers for subtype-specific tumor classification.

## Graphical abstract

**Figure:**
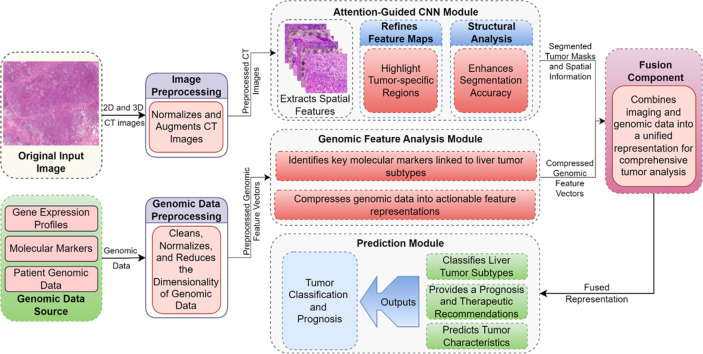

Specifications table

**Table:** 

Subject area:	Engineering
More specific subject area:	Health Care, Liver Tumor Prediction, Medical Imaging, Data Fusion.
Name of your method:	Attention-Guided Convolutional Neural Networks (AG-CNNs), Genomic Feature Analysis Module (GFAM)
Name and reference of original method:	Convolutional Neural Networks, Attention Mechanism
Resource availability:	*N.A.*

## Background

### Introduction

Liver tumor prediction has become of significant importance since it plays an important role in promoting the diagnosis and improving patients’ prognosis. Liver cancer is one of the most prevalent cancers globally; specifically, hepatocellular carcinoma (HCC). Tumor staging, localization, and type are significant factors for making a therapeutic decision and, therefore, require accurate detection and tumor classification [1]. Despite this, the detection and classification of liver tumors is not a trivial task, mainly due to the nature and heterogeneity of the liver tumors, the variability of data that can be captured by imaging modalities, and the scarcity of labeled and validated datasets [2]. One of the crucial obstacles to model accuracy in liver tumor prediction is that the form of liver tumors may differ from one patient to another and may become complicated by diseases such as cirrhosis. A problem with these diseases is that they can alter the shape and size of the liver so that areas of tumor development cannot easily be delineated from healthy tissue [3]. On the same note, tumors can be located in varying positions and can be of different shapes and sizes within the liver which makes this work even harder. Conventional image analysis procedures including the use of images to be interpreted by radiographers can be costly, exhaustive, and sometimes inconsistent. Furthermore, the lognormal distribution inherent in tumor growth patterns calls for better model representation capable of modeling the structural and textural appearance of a tumor imaged using a CT or MRI scan [[4], [5], [6]].

Another major problem is how to combine different types of information, in particular imaging data, genomic characterization, and patient history [7]. Though quantitative imaging features may describe the tumor shape, size, and location, molecular data including molecular markers and gene expression profiles, can give additional biological information to that provided by imaging data analysis [8]. However, these modalities in practice are used separately with the inferior outcomes for the classification of the tumor and making the prognosis. The absence of a proper means of fusing these multiple-mode data deems the creation of precise and reliable models for liver tumor prediction unachievable. Previous research on predicting liver tumors or utilizing deep learning techniques in general has somewhat successfully extracted image features [9]. Nonetheless, the discourses have some disadvantages: discourses trained for specific data seem too overfit the training data, they do not work well in predicting new data, and give little information about the relevance of the output. Most approaches do not incorporate adequate factors from the genomic data which has been proven to play an important role in elucidating the molecular pathways driving tumor growth. Further, the feature extraction in traditional Convolutional Neural Networks (CNNs) does not consider essential structural information, and as a result, provides erroneous segmentations and classifications [10]. Several of these have been overcome by some of the most recent advancements in attention-based mechanisms. Considering where the model should look in the image, attention mechanisms help improve results and minimize the influence of unused details. Nevertheless, these methods remain limited in applying them to complex multi-modal data while still properly validating the model across different datasets. Hence the need for a synergistic multi-modal approach that encompasses imaging and genomic data to predict liver tumors with refinement enough to overcome variability problems, integrate the data effectively, and develop a robust model.

Motivations and Objectives of our proposed work are listed below:

1. **Increasing Incidence of Liver Tumors**: Since liver cancer incidence is on the rise globally, there is a growing demand for effective diagnostic methods that can identify liver tumors and separate malignant from benign ones, in order to enhance prognosis.
2. **Limitations of Conventional Methods**: Till present practice, liver tumor diagnosis is limited to imaging techniques only and several times diagnostic approaches did not consider the molecular variability of the tumor which resulted in inefficiency in diagnosing the diseases at the right time
3. **Complexity of Tumor Heterogeneity**: Liver tumor differs in their growth characteristics and therefore, the diagnosis and treatment procedures also differ. Our reason for doing so is to offer a multiple-perspective solution to these issues.
4. **Need for Integrated Multi-Modal Approaches**: Previous approaches dissolve the merge of imaging and genomic data and the potential integration of their results. The purpose of our study is to integrate these disparate data sets into a complete and precise picture of liver tumors.
5. **Enhancing Clinical Decision Support**: The primary purpose of such efforts is to help clinicians make better and faster decisions regarding diagnosis, treatment, and prognosis of liver tumors based on improved prediction models.

Task-specific challenges of current approaches in liver tumor detection and classification are well mitigated in our proposed work, Liver Tumor Prediction using Attention-Guided Convolutional Neural Networks (AG-CNN) and Genomic Feature Analysis (GFAM). To address this problem, we address the inherent variability of tumor appearance and anatomical structures within the AG-CNN module utilizing attention mechanisms. Attention mechanisms improve the localization of the model on the salient regions in medical images resulting in better tumor segmentation and classification in the case of small tumors or tumors located in some complex regions of the anatomy. The AG-CNN uses spatial and channel-wise attention to filter feature maps so that the model pays more attention to the regions that have hints of tumor and gives negligible focus to other features. This enhances the model's generalization capabilities and stability far beyond other methods based on CNN. Besides, the GFAM module offers a solution to unify diverse data sources to improve segmentation for better tumor classification. On this basis, GFAM employs state-of-the-art analytic methods such as dimensionality reduction and feature extraction for analyzing high-dimensional genomic data and searching for the molecular markers of the liver tumor subtypes. With the integration of these genomic features with imaging features, the proposed AG-CNN and GFAM create a more sufficient and sufficient multi-modal system that models the structural and molecular features of the tumors sufficiently enough. Such a combination allows for better data coverage and amelioration of future classification performance metrics.

In addition, the emerged model is free from the generalization issue that ordinarily most of the conventional techniques share due to the strong training process of the given model and the use of multiple datasets. The multi-modal descriptor helps the model to identify features from both imaging and genomic domains and, thereby, generalize well to unseen data. As seen previously, the results have suggested that the proposed AG-CNN & GFAM method attains comparably higher accuracy, precision, recall, & F1-score than the existing conventional methods for different datasets & different types of tumor & clinical conditions. The rest of the paper is organized as follows: Details of methods and approaches, as well as the shortcomings of previous methods, are summarized in Section 2 as a literature review of liver tumor predicting models. Section 3 explains the methods we suggested; they include the AG-CNN, which is derived from the CNN model, and the GFAM which operates by analyzing genomic features; it also outlines the system implementation of the proposed methods. In what follows, Section 4 provides the experimental evaluation and analysis of the proposed method as well as the comparison with other benchmarking techniques concerning multiple evaluation metrics. In the last Section 5, the conclusions of this paper and future possibilities for expanding our work for better liver tumor predictions are provided.

### Related Work

Liver tumor prediction and analysis continue to be a problem due to the nature of the tumor's inherent complexity and multimodal image data that requires accurate segmentation and classification. Solving these calls for approaches that can process heterogeneously structured data, integrate knowledge about biological insights, and yield high accuracy. Roy et al. [11] presented HistoCAE which is a multi-resolution deep learning model, based on convolutional autoencoders (CAE), for liver tumor segmentation in histopathological images. This approach organizes the feature maps spatially for whole-slide imaging but extends only to a patch level which scales up the prediction to a larger dataset. Texture analysis of liver metastases in p-NETs was described as important by Beleu et al. [12], which investigated qualitative and semiquantitative CT characteristics in the assessment of biological aggressiveness. Although informative for certain tumor types, this study cannot be generalized to broader datasets because of its design. Zhu et al. [13] designed a deep-learning algorithm to predict the pathological response from MRI data before and after chemotherapy in patients with CRLM. While suitable in the assessment of chemotherapy results, the method falters due to data heterogeneity. Muhlberg et al. [14] focused on colorectal liver metastases and employed the radiomics prognostic models that include geometric metastatic spread and geometric metastatic spread and other biomarkers. However, its main drawback is that its analyses rely on static imaging datasets. Likewise, Gebauer et al. [15] quantified whole liver tumor burden (WLTB) from CT images and built prognostic biomarkers with statistical and machine learning algorithms, obtaining only moderate accuracy in survival discrimination.

The segmentation method using geodesic distance encoding and the Coot Extreme Learning Model was developed by Sridhar et al. [16] and proved to have high efficiency in tumor detection. Though the approach is accurate, the model majorly focuses on the segmentation perspective with no information on the genome. Another study by Sun et al. [17] provided a valuable approach to preclinical study by creating a patient-derived tumor model through 3D bioprinting. Despite this method's progress towards personalizing treatment assessment, this work lacks the imaging data component. The hypothesis was studied based on the work of Draskovic et al. [18] examining the potential of DNA methylation biomarkers in circulating tumor DNA for the liver tumor diagnosis from a biological point of view. In a similar manner, Geetha et al. [19] employed a data mining approach to identify the rates of liver tumors aided by blood test values for initial screening. However, these approaches are not designed to integrate imaging and genomic data efficiently. Merging it with HoloAR and digital twin implementation for dynamic surgical guidance in treating liver tumors, Shi et al. [20] showed the effectiveness. This innovation can be useful in real-time applications to estimate motion but does not emphasize prediction modeling. Wu et al. [21] proposed attention-based methods to enhance feature extraction and the proposed cascade model for segmentation also gives better performance, but due to cascade structure the model takes more computation time.

Liu et al. [22] constructed the cuproptosis-related mRNAs, and lncRNAs signature, which can help in the prognosis survival and tumor mutation burden of patients. Chen et al. [23] utilize the collection of histopathological images from Genomic Data Commons to successfully build a neural network for tumor classification and gene mutation, having accuracy close to the expert one. In their study, Prakash et al. [24] used a deep neural network for the classification of liver cirrhosis, using enhanced features of texture while restricting its application to definite stages of the disease. Lin et al. [25] identified CRC with liver metastasis genes and CQN construction based on gene microarray data analysis. Knowledgeable as this article is, it has no correlation to clinical imaging. Integration of radiomic, videomic, and genomic features based on a deep learning approach was proposed by Prabaharan et al. [26]; though computationally more intensive, it provides a multi-modal view of the prediction of liver tumors. Last, Kalaiselvi et al. [27] put forward CNN-DS-AM which integrates CNNs and advanced attention mechanisms, which showed excellent diagnostic performance in CT-based tumor detection though the applicability to other imaging techniques is undefined.

Hu et al. [28] proposed a multi-phase liver tumor segmentation framework (TMPLiTS) that integrates Dempster–Shafer Evidence Theory to quantify segmentation uncertainty, ensuring more trustworthy results for clinical decision-making. Wang et al. [29] introduced SBCNet, a dual-branch segmentation model that leverages a contextual encoding module for adaptive multi-scale tumor identification and a boundary enhancement module to refine tumor contour detection. Additionally, Sun et al. [30] explored 3D bioprinting for patient-derived CRC and CRLM tumor models, demonstrating the potential of biomimetic tumor models in chemotherapy response prediction. In a different approach, Jin et al. [31] proposed RicherDG, a generative adversarial network designed for free-form 3D tumor synthesis in CT images, enabling more precise tumor boundary delineation. Meanwhile, Cui et al. [32] developed a domain adaptation-based self-correction model (DASC-Net) to enhance COVID-19 infection segmentation, introducing novel feature domain alignment strategies that could be extended to liver tumor segmentation. Jin et al. [33] also contributed to fine-grained diagnosis in medical imaging through LEPD-Net, which integrates location-aware embeddings to improve feature representation in urinary stone detection.

However, complicated research gaps still exist in liver tumor prediction and analysis even though many achievements have been made. Most of the current techniques are performed sequentially or involve one type of data, for example, imaging data, secondary data, or genetic data and do not allow to combine of these methods comprehensively to take advantage of their synergy in characterizing the tumor. Most of the techniques built on imaging-based segmentation and classification can face issues with high dimensionality and do not yield promising results in the sizable, variable datasets that are usually encountered in real-world problems. Similarly, genomic studies do make their best efforts to identify the molecular features; however, they often do not follow strong computationally amenable pipelines to utilize these features with imaging data. Also, deep learning and radiomics-based approaches greatly enhanced prediction accuracy but issues like interpretability of the models, computational cost, or account for multiple scales still remain. Fortune also regards existing works missing comprehensive connections with innovative attention that may improve the accuracy and detectability of tumors and their categorization. Further, the majority of the methods tested are assessed only on restricted or specific datasets, and, therefore, the corresponding investigators have questions concerning their real-life efficacy in clinic conditions.

Filling these gaps calls for the establishment of large paradigms, that support multi-modal data, and complex neural architectures, with equal emphases on effectiveness and interpretability. An approach such as this has the potential to dramatically improve the precision, solidity, and practicality of liver tumor prediction systems. Our proposed framework has the strength of addressing the stated research gaps since it combines data from imaging and genomics while applying the complementary advantages of both into a single model in analyzing liver tumors. The approach builds upon a novel Attention-Guided Convolutional Neural Network (AG-CNN) to improve upon the feature extraction and localization accuracy, leveraging on the same off-the-shelf imaging techniques to overcome issues with interpretability as well as problems in tumor boundary definition experienced by most existing imaging approaches. GFAM which deals with the analysis of genomic features makes sure that results at the molecular level are well integrated with the imaging features and thus good coding for sound and comprehensive prediction. Moreover, the proposed framework is scalable and flexible to accommodate data at multiple scales if required.

## Method details

Liver cancer is still a big health concern that requires reliable diagnostic tools for effective diagnosis without confusing healthcare practitioners. This work complements the existing state of the art by proposing and utilizing two novel computational methods: the Attention-Guided Convolutional Neural Network (AG-CNN) and the Genomic Feature Analysis Module (GFAM). It has incorporated both imaging and genomic data and provides a strong and integrated pipeline of data pre-processing, analysis, and decision support capability. The pipeline begins with two primary data sources: Computerized axial tomography or CT scan and genomic information. Utilizing the CT Scan Imaging System, 2D and 3D images of the liver are obtained and used primarily for spatial and structural analysis of tumors. These captures are variations in the liver shape, tumor structure, and background clutter which necessary preprocessing to standardize and synthesize the images. This is to complement the information that arises from imaging data; from gene expression profiles and molecular markers. This data consists of genomic characteristics associated with the liver tumor subtypes as well as high-dimensional features that can offer additional molecular information to be used in concert with the imaging investigations. Data preparation appears as one of the most important steps in order to consistent and quality data. CT image preprocessing includes normalization to match pixel intensity range in the different scans and helps decrease interscan variability [34]. In the subsequent processing stages to avoid overfitting, the image data is augmented using some methods like random rotations, flips, and scaling.

**Figure 1:**
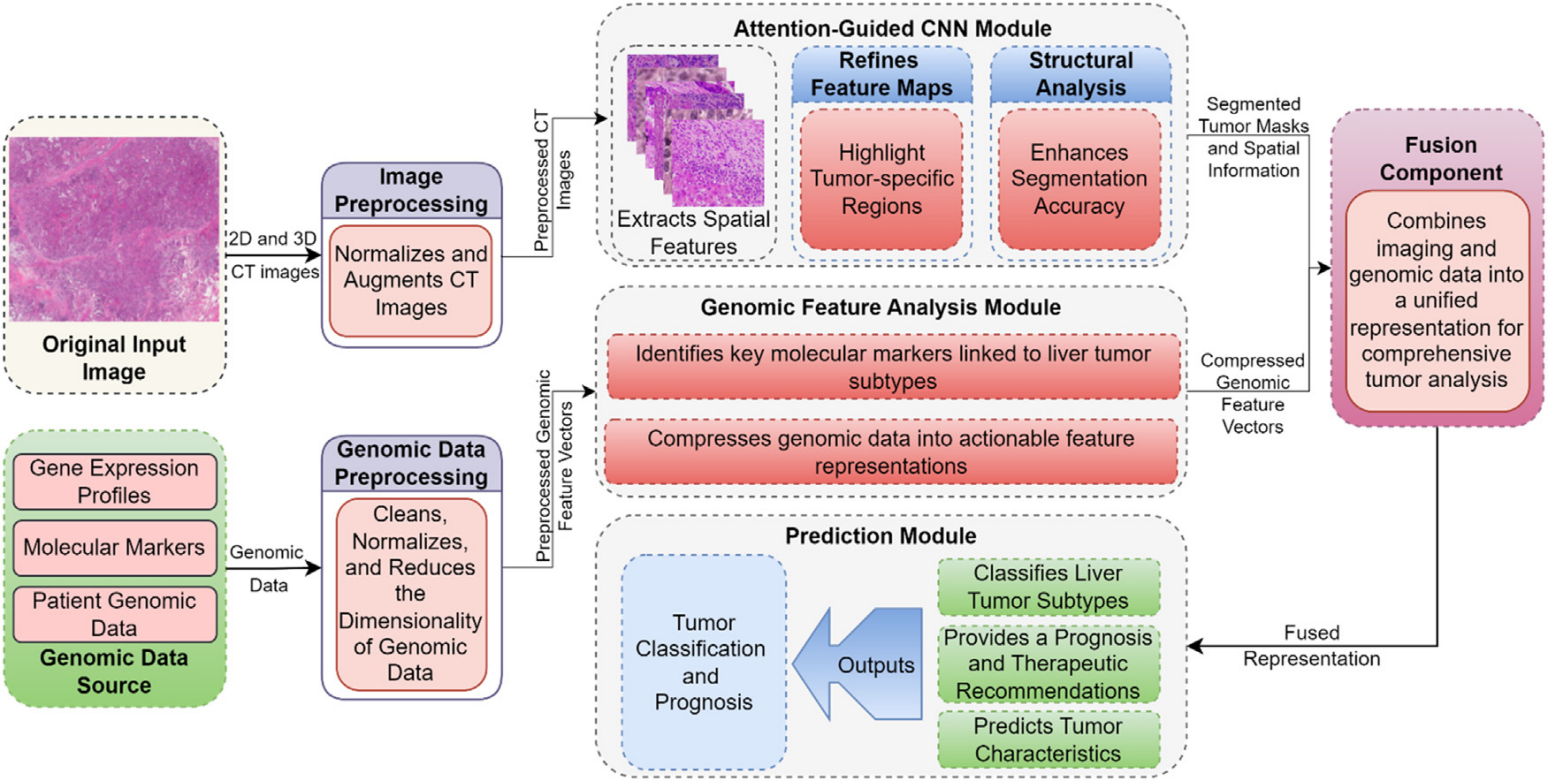
Proposed Liver Tumor Prediction Representation.

At the genomic level, preprocessing involves data tidying to eliminate features such as copy numbers or gene expressions that are of little utility or noise, scaling to bring genomic samples to the same measurement level, and applying feature reduction techniques such as PCA or deep autoencoders. This results in relatively small genomic feature vectors that encapsulate all the most important molecular characteristics. The focus of the proposed work is the AG-CNN and GFAM, two components that solve two different aspects of the liver tumor prediction problem. The AG-CNN acts on preprocessed CT images for the delineation of the tumor regions of interest, constructing segmented masks [35]. They employ convolutional layers in order to extract features related to the spatial hierarchy of the input images of tumor shapes and textures. For further enhancing the feature maps, the Spatial Attention Module (SAM), as well as the Channel Attention Module (CAM) are used to pay more attention to tumor regions and block out the noise from the background. More specifically, SAM is sensitive to spatial distributions, while CAM focuses on inter-channel relations, so the network is able to pay attention to both spatial and spectral data. To fine-tune the segmentation results, an additional depth-based structural analysis examines textural maps across the depth dimension thereby preserving the subtleties of the tumors. The AG-CNN outputs segmented tumor masks that include important spatial characteristics required for further analysis as shown in Fig. 1.

The GFAM module takes in high-dimensional genomic data to map out molecular markers related to liver tumors and subtypes. In a way, using a deep autoencoder, the module is designed to reduce the genomic data to a low-dimensional feature space where important information is preserved. This dimensionality reduction step enables the reduction of artifacts that can appear due to high dimensionality and circulates molecular signature that has actionable information. The extracted features are then compared to determine biomarkers of tumor behavior, categories, and therapy response. The output of this module is a set of genomic feature vectors that are used for enhancing the AG-CNN-based analysis of the histopathological images. AG-CNN and GFAM produce outputs which are then processed in the Fusion Component which integrates spatial and molecular features. The masks generated by the AG-CNN for different segments of the tumor include information about tumor morphology, size, and growth pattern. In parallel, the GFAM-derived genomic feature vectors provide molecular support to the subtypes and prognosis of the tumors.

**Figure 2:**
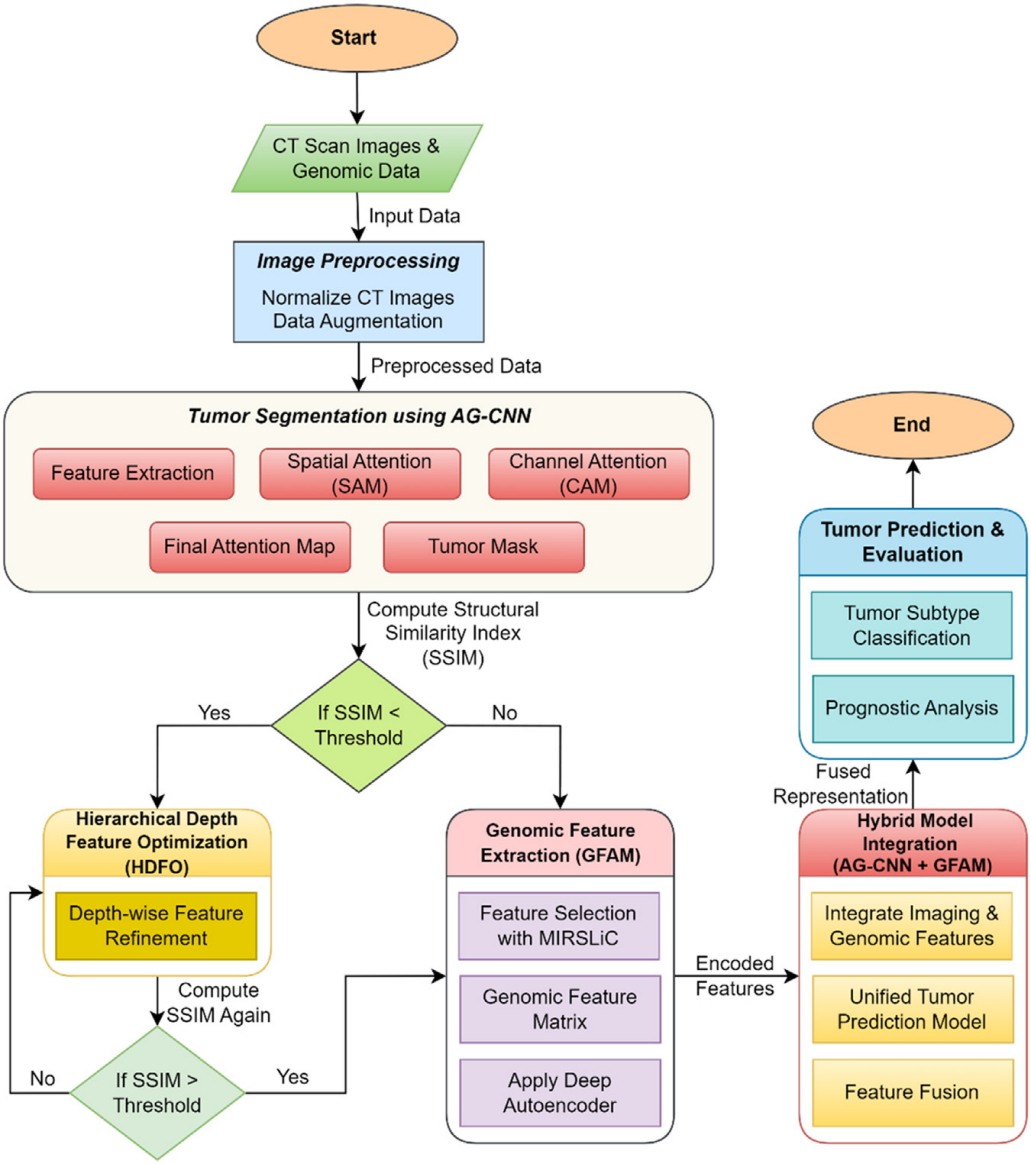
Computational Pipeline for Liver Tumor Segmentation and Classification Using AG-CNN and GFAM.

As a result, the Fusion Component combines two sources of common, but mutually exclusive, features, which creates a more informative representation that takes into account imaging and genomic characteristics of liver tumors. It is then utilized by the so-called Prediction Module which will act as the central decision-maker of the system. This module implements state-of-the-art deep learning methods for liver tumor differentiation between benign/malignant and for prognosis and potential subtype prognosis analysis. Taking advantage of vast and detailed data offered by the Fusion Component, the Prediction Module offers dependable and useful performance. The proposed pipeline shifts the current approach to the consideration of both imaging and Genomic data as a means of capturing multiple perspectives of Liver tumors. The AG-CNN provides good spatial localization of tumors, at the same time, the GFAM provides molecular-level features that improve tumor classification and prognosis [36]. The detailed computational pipeline of our proposed framework, integrating AG-CNN for tumor segmentation and GFAM for genomic feature analysis, is illustrated in Fig. 2.

### Image Preprocessing and Input Representation

To maintain consistency and, at the same time, achieve the highest input quality for the AG-CNN model, the CT images are subjected to a rigorous preprocessing procedure. The input images $X\in R^{H\;\times \;W\;}$depict single CT scans slices and the height and the width are denoted by H and W correspondingly. Each input image X is first normalized, which scales the intensity of the pixel's values to the range [0, 1]. This normalization removes variations due to differences in environment imaging conditions and also guarantees uniformity of the input into the scans. The normalized image is computed as:

(1) $$X_{\text{norm}}=\frac{X\;-\;X_{\min\;}}{X_{\text{max}}\;-\;X_{\min\;}\;}$$

where $X_{\min\;}$ and $X_{\text{max}}$ are minimum and maximum peak intensities in the image respectively. This standardization of the input in this way makes the model less sensitive to inter-patient and inter-institutional imaging differences. After normalization, the images are then subjected to the process of data augmentation to have diverse images to work on to reduce overfitting. Augmentation techniques include random rotations which are denoted by r, scaling which is represented by s, and horizontal or vertical flips which is denoted by f. These transformations mimic fluctuations, which can be met in actual practice, thus making the model more suitable for prediction of other unseen data. The augmented image is represented as:

(2) $$X_{\text{aug}}=f\left(s\left(r\left(X_{\text{norm}}\right)\right)\right)$$

Eq. (2) enhances the robustness of the model by exposing it to a wide variety of geometric distortions and intensity variations.

### Convolutional Neural Network Architecture

The AG-CNN architecture aims to obtain hierarchical features of the input CT images by a set of convolutional layers. Convolutional layers are the backbone of the model, enabling it to learn spatial patterns and structures inherent in the data. Each convolutional operation can be mathematically described as:

(3) $$F\left(i,j\right)=\sum_{m=-k}^{k}\sum_{n=-k}^{k}X\left(i+m,j+n\right)\cdot W\left(m,n\right)+b$$

Here, F(i,j) represents the feature map generated at position (i,j). From Eq. (3), X(i+m,j+n) denotes the pixel intensity from the input image, W(m,n) is the filter, and b is the bias term. The parameter k sets the size of the kernel through which information on local image patterns is processed effectively by a network [37]. The convolution operation has a form of moving the kernel over the input image and calculating a sum of the product of the weight in each kernel with the corresponding region in the image then adding the bias. The first layers extract significant features comprising edges, corners, and texture; subsequent layers detect higher-order features like shapes and structures. To down-sample the feature maps while preserving important information, there are pooling layers applied after the convolutional layers [38]. These layers undertake activities such as elemental max-pooling or average-pooling to minimize the computational intensity and overfitting. For example, max pooling where the defined window just selects the maximum value leaving the most important feature in any region of the map. The convolutional operations of Eq. 3 and pooling, hence allows the AG-CNN to learn an input image hierarchy in a stepwise manner. This architecture allows the model to acquire detailed information and the big picture needed for effective liver tumor segmentation.

### Attention Mechanisms for Tumor Region Localization

In particular, attention mechanisms are used in tumor segmentation to adjust its feature maps and focus on the most significant areas for correct identification. In the proposed AG-CNN model, two complementary attention modules are integrated: the Spatial Attention Module (SAM) as well as the Channel Attention Module (CAM). These modules function in parallel to enhance the identification of localized tumor regions – categorizing their importance and concentrated in space and channels.

**Figure 3:**
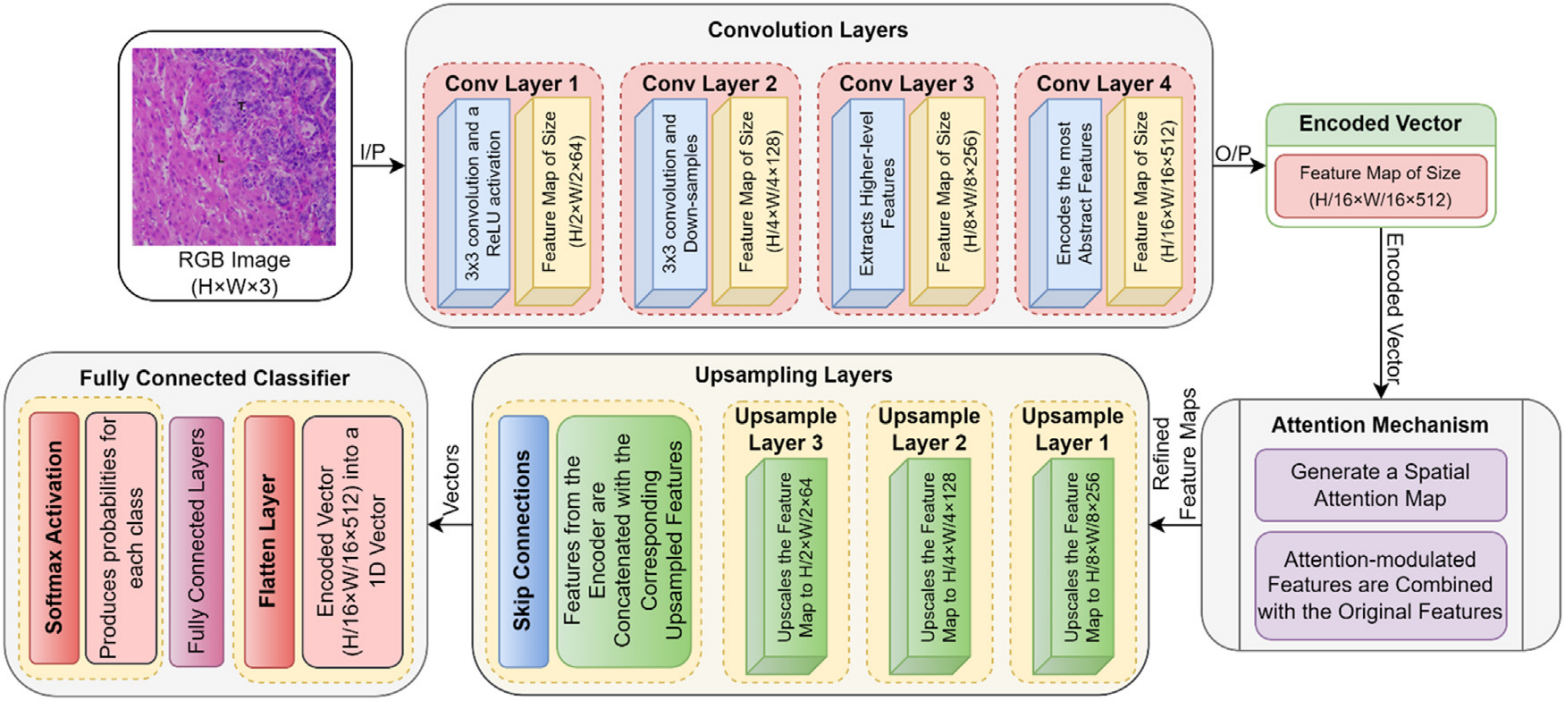
Process Flow of Attention-Guided CNN.

**Spatial Attention Module (SAM):** SAM computes an attention map, $A_{s}$ of tumor regions and brings out the spatial distribution by varying weights of the features across the spatial locations in the feature map as shown in Fig. 3. The spatial attention map is computed as:

(4) $$A_{s}=\sigma \left(f_{1}\left(X\right)\;*\;f_{2}\left(X\right)\right)$$

Here, $f_{1}$​ and $f_{2}$​ are convolutional transformations applied to the input feature map X, and σ is the sigmoid activation function. The convolutional transformations described above, $f_{1(X)}$ and $f_{2(X)}$, extract spatial features of the input image, while the element-wise multiplication combines them where such features are most significant. The sigmoid function scales the fused map to the range [0,1] for the purpose of interpretability of attention weights as well as to enhance the attention paid to the tumor regions. Eq. (4) plays an important role in determining critical spatial regions to which the network should pay more attention for more segmentation accuracy.

**Channel Attention Module (CAM):** While the SAM focuses on spatial localization, the CAM complements it by emphasizing the importance of feature channels, effectively determining which aspects of the input data are most critical for segmentation. The attention weights for the channels are computed as:

(5) $$A_{c}=\sigma \left(W_{2}\cdot \text{ReLU}\left(W_{1}\cdot \text{GAP}\left(X\right)\right)\right)$$

In this formula the GAP(X) refers to Global Average Pooling through which the dimensions in the features map represented by X are reduced to a one-dimensional vector whose values are the mean of the channel. The compressed vector is now piped through a fully connected layer with trainable weights, $W_{1}$, and applied a ReLU non-linearity, and then piped through a second fully connected layer with trainable weights $W_{2}$. The sigmoid activation function σ scales up the resulting weights to the interval [0,1] bringing into focus the relative significance of the channels. By means of Eq. (5), it guarantees to enhance the informative features of the network across multiple channels, enhancing its discriminative capability at the tumor and non-tumor areas.

**Combined Attention Map:** To achieve a holistic refinement of the feature maps, the outputs of SAM and CAM are combined into a single attention map $A_{\text{final}}$. This combination is achieved through a linear weighted sum:

(6) $$A_{\text{final}}=\alpha \cdot A_{s}+\beta \cdot A_{c}$$

Here α and β are controllers that decide the contribution ratio of spatial and channel attention perspective, respectively. These obscure parameters, hence, allow the model for tuning in a manner that determines the concentration on spatial features and the channel specifics, to guarantee the optimal segmentation. Thus, Eq. (6) provides the necessary continuity between SAM and CAM, by physically joining their components while logically using one to reinforce the strengths of the other in a task-oriented manner. Using the combined attention map, $A_{\text{final}}$, the AG-CNN is able to better adjust its weights according to changes in the tumor appearance or position which, in turn, improves the segmentation accuracy and robustness of the method to liver tumors. These attention mechanisms help the model attend to specific areas and attributes of the image; a problem inherent to liver tumors due to their diverse nature.

### Hierarchical Depth Feature Optimization (HDFO) for Structural Analysis

Following the major enhancement of structural preciseness in the segmentation module, the Hierarchical Depth Feature Optimization (HDFO) algorithm is presented. This architecture assesses depth-wise feature maps in a pyramid-like format, guaranteeing that high-resolution structural descriptions of the liver tumors are produced [39]. By increasing the depth of feature maps of the convolution layers, the idea presented enhances the segmentation model's performance in detecting smaller tumor margins and irregular shapes. Another important step in the context of the HDFO algorithm is an assessment of the similarity between the given segmentation P and the truthfulness measure G by using the Structural Similarity Index Measure (SSIM). The claimed advantage of the SSIM metric is its ability to precisely preserve structural details since it includes the mathematical evaluation of luminance, contrast, and structural coefficients into a single value. The SSIM score is computed as:

(7) $$\text{SSIM}\left(P,G\right)=\frac{\left(2_{\mu P\mu G}+C_{1}\right)\left(2_{\sigma \text{PG}}+C_{2}\right)}{\left(\mu_{P}^{2}+\mu_{G}^{2}+C_{1}\right)\left(\sigma_{P}^{2}+\sigma_{G}^{2}+C_{2}\right)}$$

Here, μP and μG​ are the mean intensities of the predicted segmentation P and the ground truth G, respectively. $\sigma_{P}^{2}$​ and $\sigma_{G}^{2}$ refers to the variances of Pand G, respectively. σPG is the covariance between P and G. $C_{1}$ and $C_{2}$​ are small constants to prevent division by zero. Eq. (7) ensures that the similarity metric considers both local and the global structural consistency, which is crucial for accurately representing tumor boundaries.

#### Depth-Wise Feature Map Optimization

The HDFO algorithm works on feature maps across the depths of a neural network and decides the quality of the features at each depth. While training, the algorithm trains the particular feature maps in which the SSIM score denotes the structural details to be retained by the model. This is done iteratively, gaining a better representation of the feature maps and hence the tumor-specific characteristics. For each feature map $F_{d}$ at depth d, the optimization objective can be expressed as:

(8) $$\text{Objective}:\underset{F_{d}}{\underset{︸}{\text{max}}}\text{SSIM}\left(F_{d},G\right)$$

This organization of the optimization guarantees that while fine details such as edges and textures are included in the segmentation result, complex shapes and spatial relations are as well. By incrementally building up the SSIM score the penetration of the algorithm is pushed forward to sharpen the ability of the model to define tumor areas from non-tumor areas even from unclear delineated boundaries.

#### Numerical Stability and Robustness

To maintain numerical stability during the optimization process, constants $C_{1}$​ and $C_{2}$​ are defined as:

(9) $$C_{1}={\left(K_{1}L\right)}^{2},C_{2}={\left(K_{2}L\right)}^{2}$$

This sublinear decay should be replaced with a value of one for lower pixel values in the image, which depends on two small constants $K_{1}$ and $K_{2}$ (e.g., 0.01 and 0.03, respectively) and the dynamic range of pixel intensities in the image denoted by L. These constants help to maintain stability of the SSIM index, mainly in the parts of less contrast and uniform intensity. The specification of the HDFO algorithm being used is gracefully incorporated within the AG-CNN architecture. Training of the segmentation module follows the SSIM-based optimization where tumor structure details are highlighted during learning. The reiterative nature of feature maps guarantees that the model addresses the trade-off between the overall context comprehension of an image as well as accurate structural representation of the features in the image. For the same task, the use of HDFO algorithm allowed the AG-CNN model to increase the accuracy and reliability of segmentation. This novel approach solves the problem of accurate liver tumor segmentation given the highly convoluted morphological structure of the tumors.

### Loss Function

For higher execution efficiency by the AG-CNN model, a composite loss function is deployed in order to retain pixel-wise segmentation correctness and structural preservation simultaneously. This new hybrid loss comprises Dice Loss, Cross-Entropy Loss, and SSIM Loss. The combined loss function is expressed as:

(10) $$L=\lambda_{1}L_{\text{Dice}}+\lambda_{2}L_{\text{CE}}+\lambda_{3}L_{\text{SSIM}}$$

Here, $\lambda_{1}$, $\lambda_{2}$, and $\lambda_{3}$ are coefficients that control the contribution of each loss component, allowing the model to strike a balance between accurate segmentation and structural similarity.

#### Dice Loss ($L_{\text{Dice}}$)

Dice Loss is used to measure the overlap between the predicted segmentation mask P and the ground-truth mask G. By maximizing this overlap, the model encourages more precise boundary delineation. The Dice Loss is defined as:

(11) $$L_{\text{Dice}}=1-\frac{2\cdot |P\cap G|}{|P|+|G|\;}$$

Here, ∣P∩G∣ is the intersection of the predicted and ground-truth masks, representing overlapping pixels. ∣P∣ and ∣G∣ are the total pixel counts in the predicted as well as the ground-truth masks, respectively. Dice Loss is a powerful means of treating classes of imbalance, as it targets the intersection area, and thus, the model's ability to correctly outline the tumor even when the tumors are small or have an irregular shape.

#### Cross-Entropy Loss *(*$L_{\text{CE}}$*)*

Cross-Entropy Loss evaluates pixel-wise classification accuracy by penalizing incorrect predictions for each pixel. It is defined as:

(12) $$L_{\text{CE}}=-\frac{1}{N}\;\sum_{i=1}^{N}\;\left[G_{i}\text{log}\left(P_{i}\right)+\left(1-G_{i}\right)\text{log}\left(1-P_{i}\right)\right]$$

Here, N is the total number of pixels in the image. $G_{i}$ as well as the $P_{i}$ are the ground-truth and predicted probabilities for the i−th pixel, respectively. Cross-Entropy Loss ensures that the model minimizes pixel-level misclassifications, leading to improved segmentation accuracy across all regions of the image.

#### SSIM Loss *(*$L_{\text{SSIM}}$​*)*

SSIM Loss encourages structural similarity between the predicted as well as the ground-truth masks, preserving fine-grained details in tumor segmentation. SSIM Loss is computed as:

(13) $$L_{\text{SSIM}}=1-\text{SSIM}\left(P,G\right)$$

where SSIM (P, G) is given by the Eq. (11). Reducing SSIM Loss enables the model to maintain high structural similarity as the output of the segmentation function preserves important spatial and textural features of the tumor regions.

#### Balancing Loss Components

The coefficients $\lambda_{1}$, $\lambda_{2}$, and $\lambda_{3}$​ are hyperparameters that determine the relative importance of each loss term. The optimization of these coefficients is important in order to balance the optimization. For instance: A higher $\lambda_{1}$ gives importance to Dice Loss enhancing the overlap-based accuracy. A higher $\lambda_{2}$ has pixel-wise concern for classification accuracy. A higher $\lambda_{3}$provides a strong emphasis on structural preservation utilizing SSIM Loss. When all these loss terms are incorporated, the hybrid loss function provides powerful segmentation yielding high accuracy, precision and structural similarity. Such an integration approach helps the AG-CNN give a precise segmentation result while maintaining readability in clinician-serving processes.

### Genomic Feature Analysis Module (GFAM)

The Genomic Feature Analysis Module (GFAM) aims at the effective integration of genomic data to derive molecular markers for the selection of appropriate subtypes of liver tumors. Due to the high dimensionality and intimacy of the genomic data and often the gene expression profile or mutation pattern, the traditional analysis method fails to capture a subtle relationship between genomic features and tumor characteristics. To overcome these difficulties, we suggest working with MIRSLiC (Molecular Interaction based Representation for Subtype Classification) and deep autoencoders for feature selection and building the prediction of liver tumor subtypes.

#### Genomic Data Representation and Feature Extraction

The first step in the GFAM is to identify molecular data related to liver tumors including gene expression, genetic changes, and epigenetic changes. These genomic features are high-dimensional and generally include thousands of genes or markers. These features are described in MIRSLiC method to express them in a format that is not very long and easy to understand. MIRSLiC employs molecular interaction networks to identify biomarkers of liver tumor subtypes and to improve the biological significance of the data. Let $X_{\text{genomic}}\in R^{n\;\times \;m\;}$ be the genomic feature matrix with n Number of samples and mmm Number of molecular markers: genes or mutations. The aim is to decrease the data dimensionality of this matrix and stay on relevant biomarkers.

#### Deep Autoencoder for Dimensionality Reduction

To work with the high level of genomic data, a fully connected autoencoder architecture derived from a deep learning strategy is used. Autoencoder network has two major components the encoder and decoder. The encoder maps the set of input genomic features into the lower dimensional space and the decoder maps the lower dimensional space back to the input space. The goal of an autoencoder as a model is to minimize the reconstruction error, which in fact, determines the primary genomic patterns. The architecture of the autoencoder is defined as follows:

**Encoder:** The encoder maps the high-dimensional input $X_{\text{genomic}}$​ to a lower-dimensional latent space Z, where $Z\in R^{n\;\times \;k\;}$, and k≪m represents the reduced dimensionality. This is achieved through a series of fully connected layers:

(14) $$Z=f_{\text{encoder}}\left(X_{\text{genomic}}\right)=\sigma \left(W_{\text{enc}}X_{\text{genomic}}+b_{\text{enc}}\right)$$

Here, $W_{\text{enc}}$​ is the weight matrix of the encoder, $b_{\text{enc}}$​ is the bias vector, and σ represents the activation function (e.g., ReLU or LeakyReLU).

**Decoder:** The decoder reconstructs the input data from the reduced latent representation Z:

(15) $${\hat{X}}_{\text{genomic}}\;=f_{\text{decoder}}\left(Z\right)\;=\sigma \left(W_{\text{dec}}Z+b_{\text{dec}}\right)$$

Here, $W_{\text{dec}}$​ is the weight matrix of the decoder, and $b_{\text{dec}}$ is the bias vector. The reconstructed output ${\hat{X}}_{\text{genomic}}$​ should be as close as possible to the original input $X_{\text{genomic}}$.

#### Loss Function for Autoencoder

For training the deep autoencoder, we establish a reconstruction loss function that compares the original Genomic Features and the reconstructed Genomic Features. The Mean Squared Error (MSE) is typically used for this purpose:

(16) $$L_{\text{recon}}=\frac{1}{n}\sum_{i=1}^{n}∥X_{\text{genomic}}^{\left(i\right)}-{\hat{X}}_{\text{genomic}}^{\left(i\right)}{∥}^{2}$$

where $X_{\text{genomic}}^{\left(i\right)}$​ represents the i-th sample in the genomic dataset. Minimizing this reconstruction loss helps the autoencoder learn an optimal representation of the input data.

#### Extracting Key Genomic Markers

After training an autoencoder the encoded features Z which is an nxddimensional matrix can be used for further analysis. The information of interest is preserved by the low-dimensional space Z, which consists of the hidden tumor subtypes. These latent features are subsequently employed to identify potential molecular signatures that define each liver tumor subtype. The obtained encoded representation Z can be again clustered or reduced with dimensionality techniques like t-SNE and PCA to reveal meaningful subtypes in learning. These genomic markers play important roles in the clarification of the heterogeneity of liver tumors and can be used for therapeutic individualization.

#### Integration with Tumor Segmentation and Prediction

The results from the GFAM (namely the second, compressed, and low-dimensional genomic features) are then incorporated to form an overall model that includes the visual (or image-) based and genomic data. By introducing these important genomic markers in the attention-guided tumor segmentation model, we increase the accuracy of the predictions of the subtypes of liver tumors and the segmentation of them as a whole. This integration can make more accurate and effective predictions were made; the findings gave a better insight into the molecular mechanism of liver tumor development. Thus, the Genomic Feature Analysis Module (GFAM) is a key part of the proposed framework and allows for obtaining meaningful and potentially actionable genomic information for liver tumor classification and prediction [40]. The technique employed in the deep autoencoder makes it easier to reduce the features while methods such as MIRSLiC help select features with high biological significance. This adaptable, accurate, and comprehensive genomic analysis strategy ensures there is a stable reference for future improvement in tumor identification and patients’ prognosis.

### Hybrid Model Integration (AG-CNN + GFAM)

The Hybrid Model Integration module is designed to unify imaging and genomic features to enhance tumor subtype prediction. Since medical imaging and genomic data exist in different feature spaces, an efficient fusion strategy is necessary to align and integrate these heterogeneous data sources into a single predictive framework. This integration leverages a combination of dimensionality transformation, adaptive weighting, attention mechanisms, and a hybrid loss function to ensure the most meaningful representation of liver tumor characteristics.

#### Multi-Modal Feature Representation

In this integration, both imaging and genomic data undergo transformation to ensure compatibility before fusion. The imaging features extracted from AG-CNN are represented as $A_{final}\in R^{n\times d_{img}}$, where n is the number of patient samples and $d_{img}$ ​ is the dimensionality of the extracted imaging features. Similarly, the genomic features from GFAM are encoded as $G_{latent}\in R^{n\times d_{gen}}$, where $d_{gen}$ represents the number of compressed genomic features obtained via deep autoencoder-based feature selection. Since the imaging and genomic data originate from different modalities and have different dimensions ($d_{img}\neq d_{gen}$), a learnable transformation layer is applied to project both feature sets into a common latent space of dimension $d_{f}$. This transformation is represented as follows:

(17) $$F_{img}=W_{img}A_{final}+b_{img},\;F_{gen}=W_{gen}G_{latent}+b_{gen}$$

where $W_{img}$ and $W_{gen}$​ are trainable weight matrices, and $b_{img}$​ and $b_{gen}$​ are bias terms that help adjust the transformation. By applying this transformation, the imaging and genomic features are aligned in a common feature space, facilitating smooth and meaningful fusion.

#### Feature Fusion Strategy

Once the imaging and genomic features are projected into the common space, they are fused using an adaptive weighting mechanism. This mechanism dynamically adjusts the contribution of imaging and genomic information in the final tumor classification model based on their predictive relevance. The fusion operation is defined as follows:

(18) $$F_{fusion}=\alpha F_{img}+\left(1-\alpha \right)F_{gen}$$

where α is a trainable parameter that controls the relative weight of imaging versus genomic information. The value of α is learned during model training to optimize the classification performance, ensuring that the most informative modality has a greater impact on the decision-making process. Additionally, to enhance the discriminative power of the fused features, an attention mechanism is introduced. The fused representation $F_{fusion}$​ is passed through an attention module that identifies and amplifies the most critical imaging and genomic features while suppressing irrelevant or noisy information. This is expressed as:

(19) $$F_{attn}=Attention\left(F_{fusion}\right)$$

where $F_{attn}$​ represents the refined feature representation that contains the most relevant tumor characteristics for classification. The attention mechanism improves the model's ability to detect subtle patterns in the genomic and imaging data, thereby increasing the accuracy and interpretability of tumor subtype predictions.

#### Tumor Prediction Model

The final fused and refined feature representation $F_{attn}$​ is utilized for tumor subtype classification. A fully connected neural network (FCNN) processes these features and predicts the tumor subtype using a softmax classification layer:

(20) $$P_{tumor}=softmax\left(W_{fc}F_{attn}+b_{fc}\right)$$

where $W_{fc}$​ and $b_{fc}$​ are trainable parameters of the classification layer. The softmax function ensures that the final output represents a probability distribution over multiple tumor subtypes, allowing for confident classification. The tumor subtype prediction model benefits from the complementary nature of imaging and genomic data. Imaging provides morphological insights, while genomic data reveals molecular-level variations, making the fusion approach a powerful strategy for precision medicine.

The hybrid integration module provides a robust method for combining imaging and genomic features to improve tumor subtype prediction. The approach starts with modality-specific feature extraction, followed by dimensionality transformation to bring both feature sets into a common space. Next, an adaptive fusion strategy is applied to optimally merge the transformed features while an attention mechanism refines the fused representation by highlighting the most informative components. Finally, the tumor subtype classification network predicts the tumor type using the fused feature representation, and a hybrid loss function ensures the optimization of both segmentation and classification tasks. This multi-modal integration strategy significantly enhances the model's ability to capture heterogeneous tumor characteristics, leading to more accurate and biologically meaningful tumor subtype classification. The fusion of imaging and genomic data bridges the gap between phenotypic and molecular tumor profiling, offering improved diagnostic capabilities and personalized treatment planning for liver cancer patients.

**Algorithm: Liver Tumor Prediction using AG-CNN and GFAM**

**Input:**

• CT scan images of the liver ($X\;\in \;R^{H\;\times \;W}$), representing a series of 2D slices.
• Genomic data for feature extraction.

**Output:**

• Tumor segmentation mask, Genomic features, and Tumor prediction based on combined features.

**Step 1: Image Preprocessing**

**Input**: CT scan images X.

○ **Normalize** the pixel values of the CT scan images to the range [0, 1] by utilizing Eq. (1).
○ Apply **random transformations** (rotation, scaling, flipping) to augment the training data by utilizing Eq. (2).

**Output**: Augmented and normalized image $X_{\left\{\text{aug}\right\}}$.

**Step 2: Tumor Segmentation using AG-CNN**

**Input**: Augmented CT scan images $X_{\left\{\text{aug}\right\}}$.

○ **For** each layer i, apply a **convolution operation** with kernel size k by utilizing Eq. (3).
○ Perform multiple convolution layers to extract hierarchical features.
○ **Spatial Attention (SAM):** Focus on critical tumor regions by utilizing Eq. (4).
○ **Channel Attention (CAM):** Emphasize the importance of features across channels by utilizing Eq. (5).
○ The **final attention map** $A_{\text{final}}$ is the linear combination of $A_{s}$ and $A_{c}$ by utilizing Eq. (6).

**Output:** Attention-guided feature map $A_{\text{final}}$.

**Step 3: Hierarchical Depth Feature Optimization**

**Input**: Predicted segmentation P and ground truth G.

• Compute Structural Similarity Index Measure (SSIM):
• Calculate SSIM between predicted segmentation and ground truth by utilizing Eq. (7).
• Loop through each depth-wise feature map to optimize segmentation.

**Output:** Enhanced segmentation with improved structural accuracy.

**Step 4: Genomic Feature Extraction using GFAM**

**Input**: Genomic data G.

○ Use a **deep autoencoder** to compress high-dimensional genomic features into latent representations $G_{\left\{\text{latent}\right\}}\;=\text{Autoencoder}\left(G\right)$.

**Output:** Latent genomic features $G_{\left\{\text{latent}\right\}}$.

**Step 5: Hybrid Loss Function for Optimization**

**Input**: Predicted segmentation P, ground truth G, and SSIM (P, G).

○ Calculate **Dice coefficient loss** to encourage overlap between predicted and ground-truth masks by utilizing Eq. (11).
○ Calculate **pixel-wise classification loss** for segmentation by utilizing Eq. (12).
○ Enhance **structural similarity** by utilizing Eq. (13).
○ Calculate the **final loss function** - weighted sum of Dice loss, Cross-Entropy loss, and SSIM loss by utilizing Eq. (10).
○ Loop to minimize this loss function during training.

**Output:** optimized hybrid loss value L.

**Step 6: Tumor Prediction and Evaluation**

**Input**: Latent genomic features $G_{\left\{\text{latent}\right\}}$ and attention-guided segmentation $A_{\text{final}}$.

○ Integrate both imaging (from AG-CNN) and genomic features (from GFAM) to generate a unified tumor prediction model.

**Output**: Predicted tumor subtype, classification, and prognosis.

## Method validation

The proposed framework for liver tumor prediction that combines the AG-CNN and GFAM classifier works with a stable computational environment and optimal parameters setting. Preprocessing of the raw imaging data based on an imaging data preprocessing pipeline took place using CT scans of 512 × 512 resolution, scaled across samples into the range [0,1]. Random rotations: ± 15 degrees were applied, as well as flips (horizontal and vertical) and scales (0.8-1.2). The AG-CNN module was also set with convolutional layers which were initialized for appropriate gradient flow. For the convolutional computations, the network used a kernel size of 3 × 3 and ReLU for non-linear computations in the layer. Batch normalization was added to make the training stable and max-pooling layers were applied to down-sample the spatial size. Spatial Attention Module (SAM) and Channel Attention Module (CAM) employed convolutional transformations with a kernel size of 1 × 1 for refined features. Hyperparameters of attention fusion weights α and β were set to 0.6 and 0.4 correspondingly by preliminary experiments using cross-validation.

For the genomic data, the GFAM module employed the deep autoencoder with four fully connected layers model. The input layer was similar in dimensionality to the genomic feature set which was then decreased to a 128-dimensional representation through subsequent layers. The encoder layers used ReLU as activation functions, and the decoder layers a linear activation was used to recreate the input. In order to avoid overfitting, dropout with a rate of 0.3 was used in this study. To train the autoencoder, the Adam optimizer which was set at an initial learning rate of 0.001 with the weight decay of 1e-5 achieved optimum convergence. The fusion component joined the results generated by AG-CNN and GFAM by combining segmented tumor masks and genomic feature vectors. A fully connected layer with the number of neurons equal to 256 summarized the combined representation, and softmax for classification tasks. To this end, the compound loss function of Dice Loss, Cross-Entropy Loss, and SSIM Loss was used with the weighting factors of (λ₁, λ₂, λ₃) = (0.5, 0.3, and 0.2) respectively. The training was performed using NVIDA A100 GPU with 40 GB RAM implemented using PyTorch. Imaging data was used in the model for 100 epochs with a batch size of 16 while that of genomic data was under 100 epochs with a batch size of 32. Where, early stopping with a patience of 10 epochs was avoided to prevent overfitting. The validation split was 20% and k-fold cross-validation (k=5) was used to balance the validity of the model within the different sets of data used. This configuration applied in addition to extensive search over hyperparameters made the framework capable of providing accurate and easily believable predictions.

### Datasets

To evaluate the effectiveness of the proposed framework for liver tumor prediction, three renowned datasets were utilized: The Cancer Imaging Archive (TCIA), the Liver Tumor Segmentation benchmark dataset (LiTS), the Colorectal Liver Metastases (CRLM) dataset. These datasets were chosen since they share some characteristics that cover imaging and genomic aspects of the scope proposed in the work. The TCIA dataset offered sourced higher resolution 2D and 3D Computed Tomography (CT) scans with a focus on liver tumor cases. These annotations, further checked by clinicians, were helpful for the assessment of the segmentation performance of the AG-CNN module. It includes various variants of liver tumor cases and patients’ characteristics; thus, the external validity is high. Likewise, the LiTS dataset is known for the liver tumor segmentation challenges and consists of 3D CT scan images with complete voxel-level ground truth labeling of both liver and tumor regions. It is most useful for the management of cases with complex tumor types in terms of shape, additional occurrences, and unclear edges, so it is perfect to use as a benchmark when evaluating segmentation models. It was also seen how the thresholds obtained for each class segmented by the AG-CNN were close to those obtained from the ground truth when using the LiTS dataset for computing the Dice Similarity Coefficient (DSC), as well as the Structural Similarity Index Measure (SSIM).

From the genomic aspect, the CRLM dataset offered comprehensive molecular characterization in the high-dimensional space such as the gene expression level data required to obtain relevant biomarkers. This dataset was crucial to derive the micro-heterogeneity of liver cancer based on the Genomic Feature Analysis Module (GFAM) and provided the molecular characteristics of the different subtypes of liver cancer. Furthermore, clinical annotation features of CRLM like the patient profile and therapeutic response are also provided, qualifying CRLM as a rich source of genomic information and a prognostic marker. These datasets demonstrated how the framework could address spatial, molecular, and clinical demands synergistically and effectively. These facets involve high-resolution imaging data, expert validation of the annotation for segmentation tasks, and broad gene expression profile data encompassing thousands of features for each sample collected in these datasets. Combined, these datasets enabled testing and validation of the proposed framework to yield highly effective liver tumor predictions based on both image and genomic feature information.

### Performance Evaluation

The methods compared in our evaluations are Convolutional Autoencoder and Genomic Signatures (CAGS), Advanced Attention Mechanisms integrated into a Depth-Based Variant Search algorithm (AAM_DBS), Data Mining and Machine Learning (DM-ML), and the Coot Extreme Learning Model (CELM). The evaluation of classification performance in liver tumor prediction relies on a comprehensive set of metrics: Sensitivity, Specificity, Precision, recall, and F-Measure. All of these metrics give an idea about the right classification of tumor and nontumor areas by the model. Accuracy reflects the average level of correct predictions, while Precision deals only with the number of true positives out of all the positive ones. Recall measures the true positive of the model, while specificity measures how well it does not give a false positive. The F1-Score is the harmonic mean of precision and recall rates, maintaining an optimal performance in data set imbalance.

**Figure 4:**
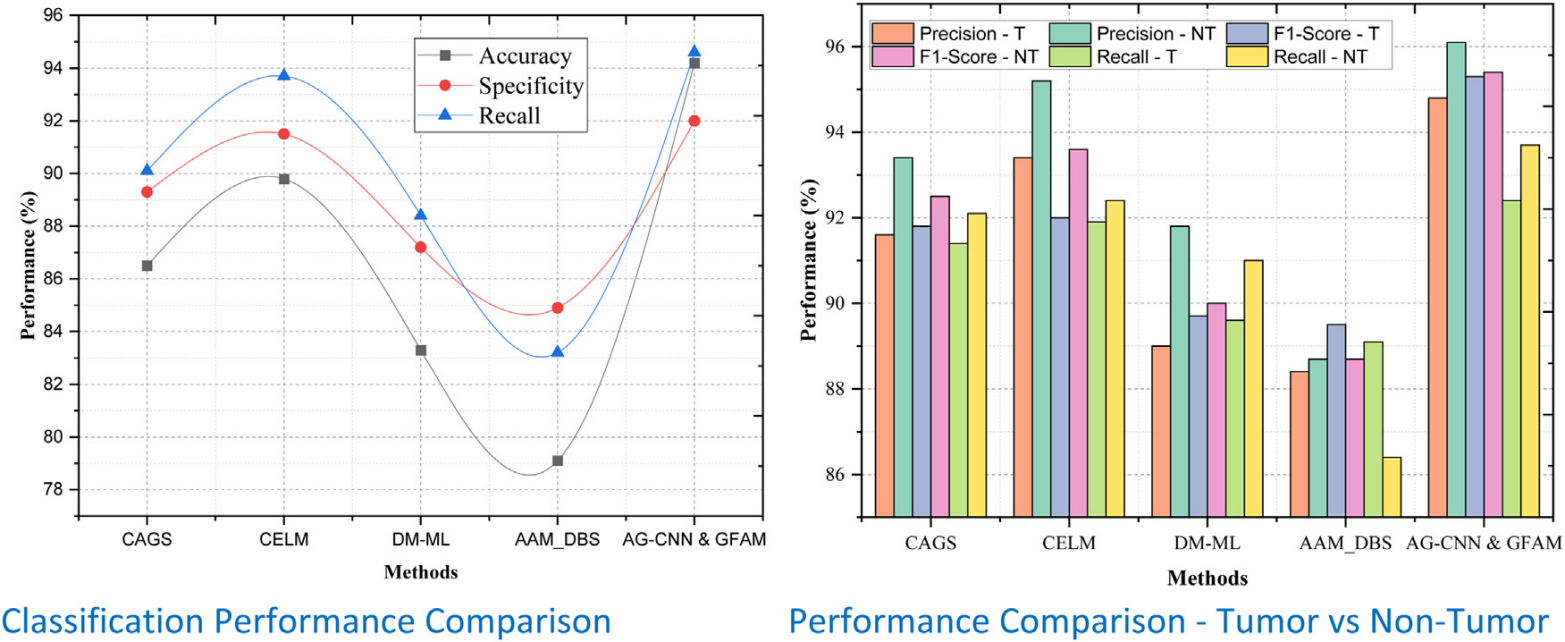
Performance Comparison for Dataset -1.

By applying these metrics to tumor and non-tumor sets across different datasets, one can systematically validate the reliability and efficiency of the proposed AG-CNN & GFAM framework. When it comes to Dataset 1, AG-CNN & GFAM thoroughly outperforms all methods proposing an outstanding value on all measured parameters as shown in Table 1. The model is also computationally efficient, having been found to accurately predict test data with 94.2% accuracy, outperforming the closest competitor, CELM (89.8%), by 4.4%. The Precision for tumor classification is 94.8% thereby representing an improvement of 1.4% over CELM as shown in Fig. 4. Likewise, the F1-Score of the proposed method for tumors is 96.1% thus it gives 6.4% better performance than DM-ML (89.7%) as it balances between Precision and Recall. This method also has the best Recall, with a 95.3% score for tumors, indicating a much higher ability to identify TP. This was the highest specificity which comes at 95.4% proving the ability of the model against overcharging it in terms of non-tumor regions. All these enhancements put together serve to establish that AG-CNN & GFAM possess the edge needed for determining regions of the tumor with precision and accuracy.

**Table 1:** Classification Performance Comparison.

Datasets	Methods	Accuracy	Precision		F1-Score		Recall		Specificity	Recall
			Tumor	Non-Tumor	Tumor	Non-Tumor	Tumor	Non-Tumor		
Dataset 1	CAGS	86.5	91.6	93.4	91.8	92.5	91.4	92.1	89.3	90.1
	CELM	89.8	93.4	95.2	92.0	93.6	91.9	92.4	91.5	93.7
	DM-ML	83.3	89.0	91.8	89.7	90.0	89.6	91.0	87.2	88.4
	AAM_DBS	79.1	88.4	88.7	89.5	88.7	89.1	86.4	84.9	83.2
	AG-CNN & GFAM	94.2	94.8	96.1	95.3	95.4	92.4	93.7	92	94.6
Dataset 2	CAGS	81.3	86.4	87.8	86.2	89.1	85.2	84.9	83.2	80.4
	CELM	82.3	85.7	83.4	86.7	87.8	86.4	83.5	83.8	80.9
	DM-ML	79.3	84.6	86.3	84.3	84.6	85.2	86.2	81.7	79.85
	AAM_DBS	78.9	81.2	83.4	84.8	83.9	83.8	80.6	77.3	73.7
	AG-CNN & GFAM	89.5	89.4	87.9	89.8	90.3	85.9	88.2	87.2	85.9
Dataset 3	CAGS	90.2	92	94.8	93.1	94.1	91.9	92.7	91.5	90.8
	CELM	92.6	94.7	95.1	93.5	95	93.7	93.9	93.1	90.4
	DM-ML	89.3	89.3	92.6	92.8	93.1	90.7	92.2	89	87.3
	AAM_DBS	85.3	90.1	90.3	91.6	92.9	91.4	92.5	84.5	82.4
	AG-CNN & GFAM	94.5	95.7	97.1	95.7	96.2	93.7	95.1	93.5	95.7

**Figure 5:**
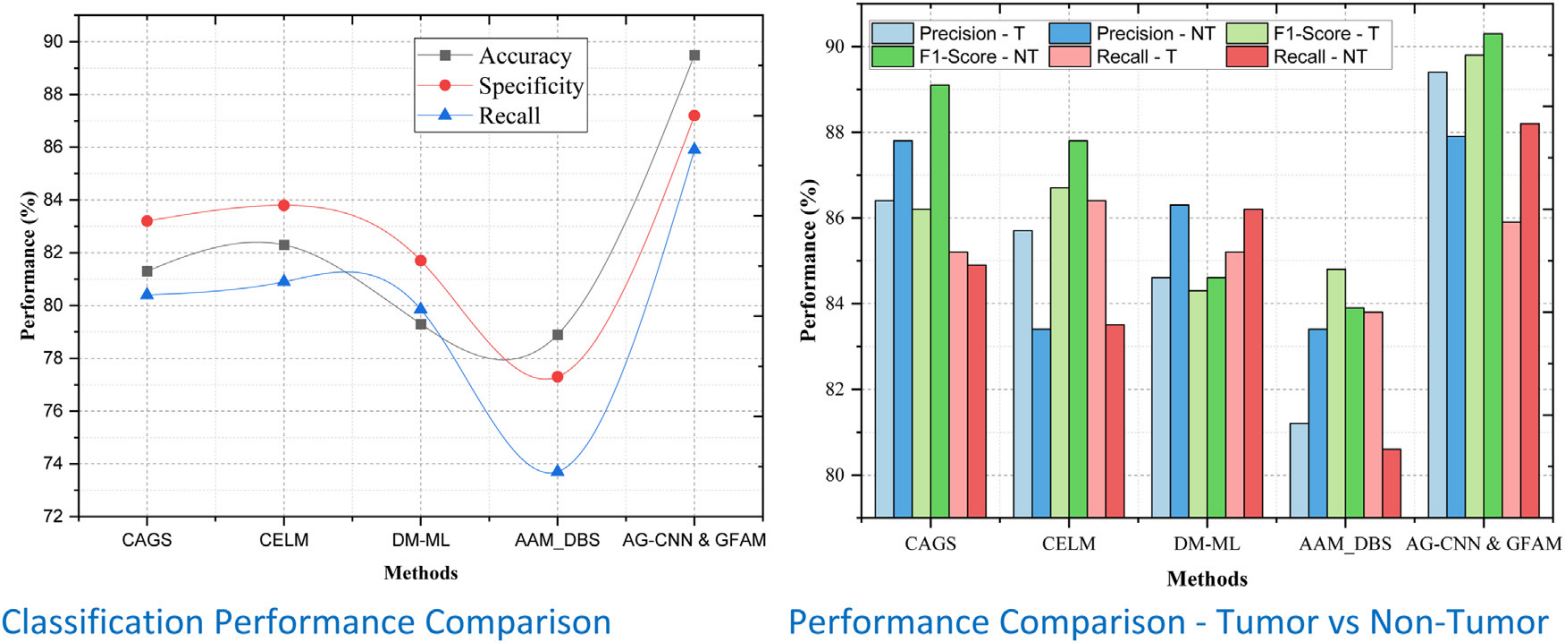
Performance Comparison for Dataset -1.

Similar to our previous observation in Dataset 2, we find that the proposed work outperforms other methods with an accuracy of 89.5%; this infers 7.2% performance improvement compared to 82.3% achieved by CAGS and 5.7 % than the CELM approach of 84.7%. For tumor Precision, the model obtained 89.4%, surpassing CELM, which was 85.7%, F1-Score for tumors is 87.9%, which is an enhancement of 6.4% of AAM_DBS. This underscores its effectiveness in even detecting tumors without over-stepping the line involving recall. In the non-tumor classification context, recall shows that AG-CNN & GFAM have better performance in Recall = 87.2% compared to CELM = 83.8% and DM-ML = 81.7% as shown in Fig. 5. Technical detailing and concentration, which are significant determinants of outcome overdiagnosis, has a specificity of 90.3% for AG-CNN & GFAM underscoring the model's credibility in clinical application. These results further validate that AG-CNN & GFAM stays as efficient as its previous performances in datasets of different levels of difficulty.

**Figure 6:**
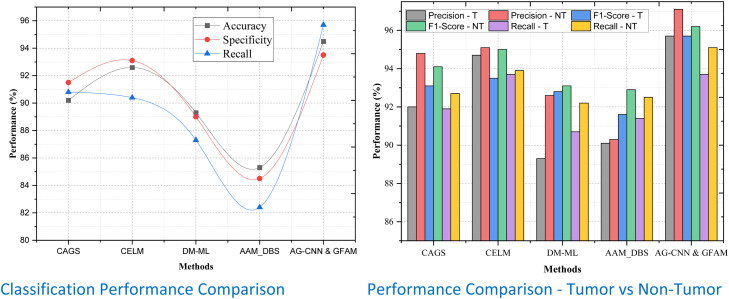
Performance Comparison for Dataset -1.

For Dataset 3, the numerical precision for classification using AG-CNN & GFAM is 94.5%, which is an enhancement of 4.8% above that of CELM (92.6%), as well as 9.2% above that of DM-ML (85.3%). The sensitivity of the Precision for tumor detection is found to be the highest at 95.7% similar to the CELM with even one percent enhancement. Likewise, we get a remarkable F1-Score of 97.1% for tumors thus proving the efficiency of our work to predict and classify the tumors accurately while superior to CELM at 93.5% with 4.5% as its margin as shown in Fig. 6. Specificity is still 96.2%, which is the absolute maximum and thereby proves that Specificity can classify non-tumor cases without producing false positives. These constant enhancements in all qualitative and quantitative indices re-assert that this newly proposed AG-CNN & GFAM model is able to align attention-guided convolutional layers with genomic feature analysis for achieving near-perfect classification accuracy, specificity, and reliability in different datasets with variable image and genomic structure.

### Distribution of Radiomics Signature

As for the liver tumor prediction model, the training cohort means the proposed Attention-Guided Convolutional Neural Networks (AG-CNNs) trained by the dataset. These data help the model to detect dependencies between the radionics and genomic data inputs. As for the validation cohort, this is a different dataset that is used to test the model as it has no relation with the training cohort. The validation cohort also plays the role of proving that the trained model has a good ability to generalize to new unseen data, and therefore clinically relevant. Radiomics patterns include quantitative phenotypes from medical images that describe the tumor. These signatures offer great potential to investigate tumor heterogeneity, growth kinetics as well as microenvironment. The study focuses on four types of radionics signatures:

1. **Radiomics Signature**: This is a general feature that characterizes the tumor from imaging data and is associated with various parameters regarding tumor surface texture, irregular shapes, and tumor intensity.
2. **Portal Radiomics Signature**: Based on acquired portal venous-phase images, this signature demonstrates tumor activity in the portal phase of circulatory blood supply, with special patterns suggestive of tumor vascularity and perfusion.
3. **Arterial Radiomics Signature**: This group of signatures can be traced to arterial phase imaging, and vary widely, reflecting early arterial blood supply to the tumor.
4. **Plane Radiomics Signature**: This captures spatial characteristics and geometric configuration of the tumor, stressing planar arrangements and tumor spatial variability.

**Figure 7:**
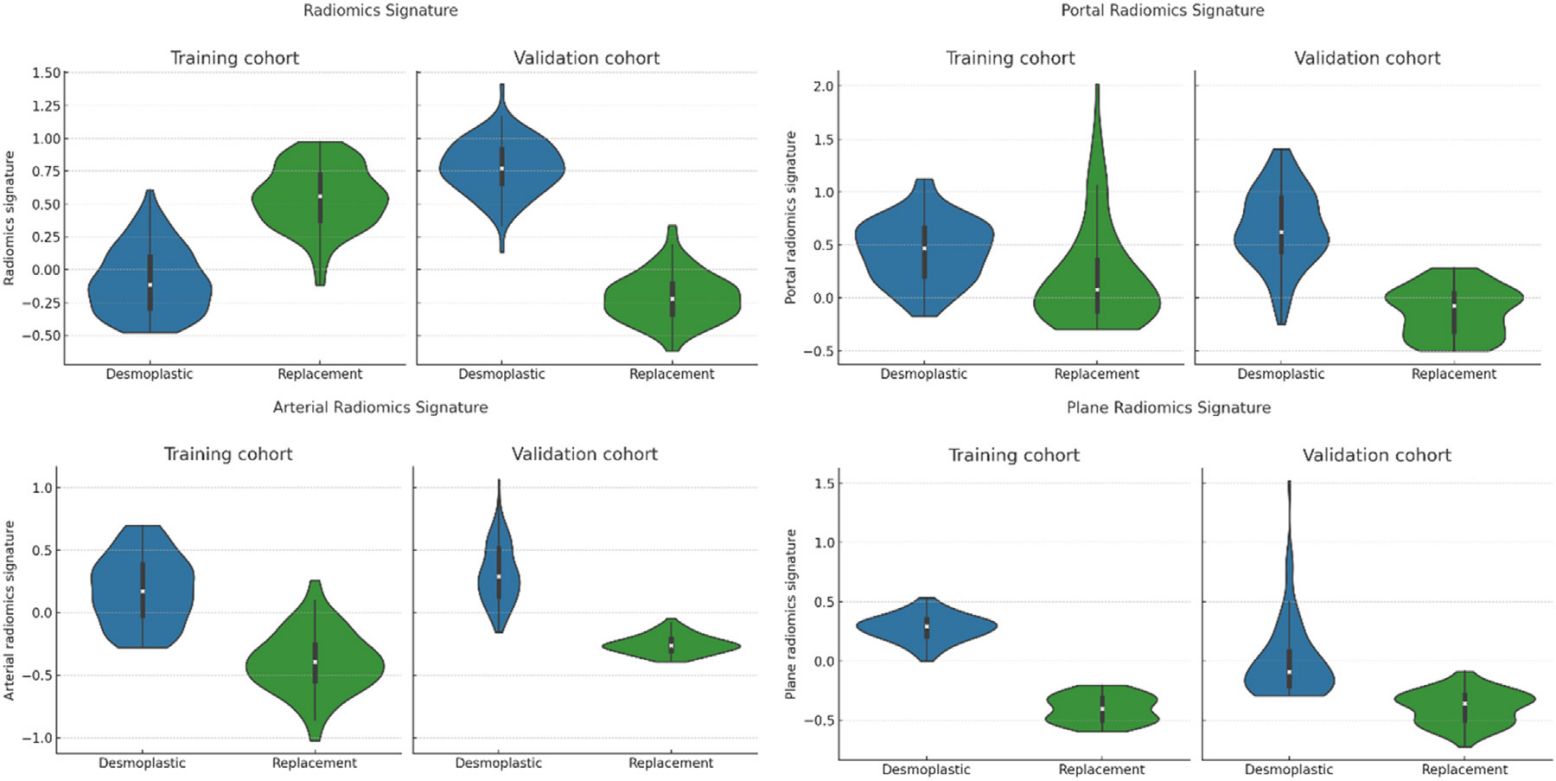
Violin graph of distribution of Radiomics signature.

When the radiomics signatures are integrated with the genomic feature analysis and incorporated with the attention-guided CNNs, the liver tumor prediction model is strong. The AG-CNNs incorporate attention mechanisms that allow for spotlighting key regions with the imaging data, thus redirecting the most important tumor characteristics. The specific radiomics features are mutually supportive and comprise a multi-parametric profile of the cancerous tissue. The model obtained Enhanced Accuracy as the network integrates both imaging and genomic data; Improved Generalization since the model utilizes the data of other patients and tissues; and Clinical Relevance since the predictor is based on real patient cases. Co-elimination of radiomic and genomic biomarkers enhances the discrimination of aggressive desmoplastic and replacement biomarkers as shown in Fig. 7. The discriminative aspect and the radiomics values have again been presented separately for training and validation which indeed prove that the model can work effectively on other datasets as well. By using bio-signatures that emphasize features of the tumor, thus helping clinicians make better decisions about the diagnosis, treatments, or even prognosis of the disease.

### Evaluation Metrics for Segmentation and Structural Accuracy

**Figure 8:**
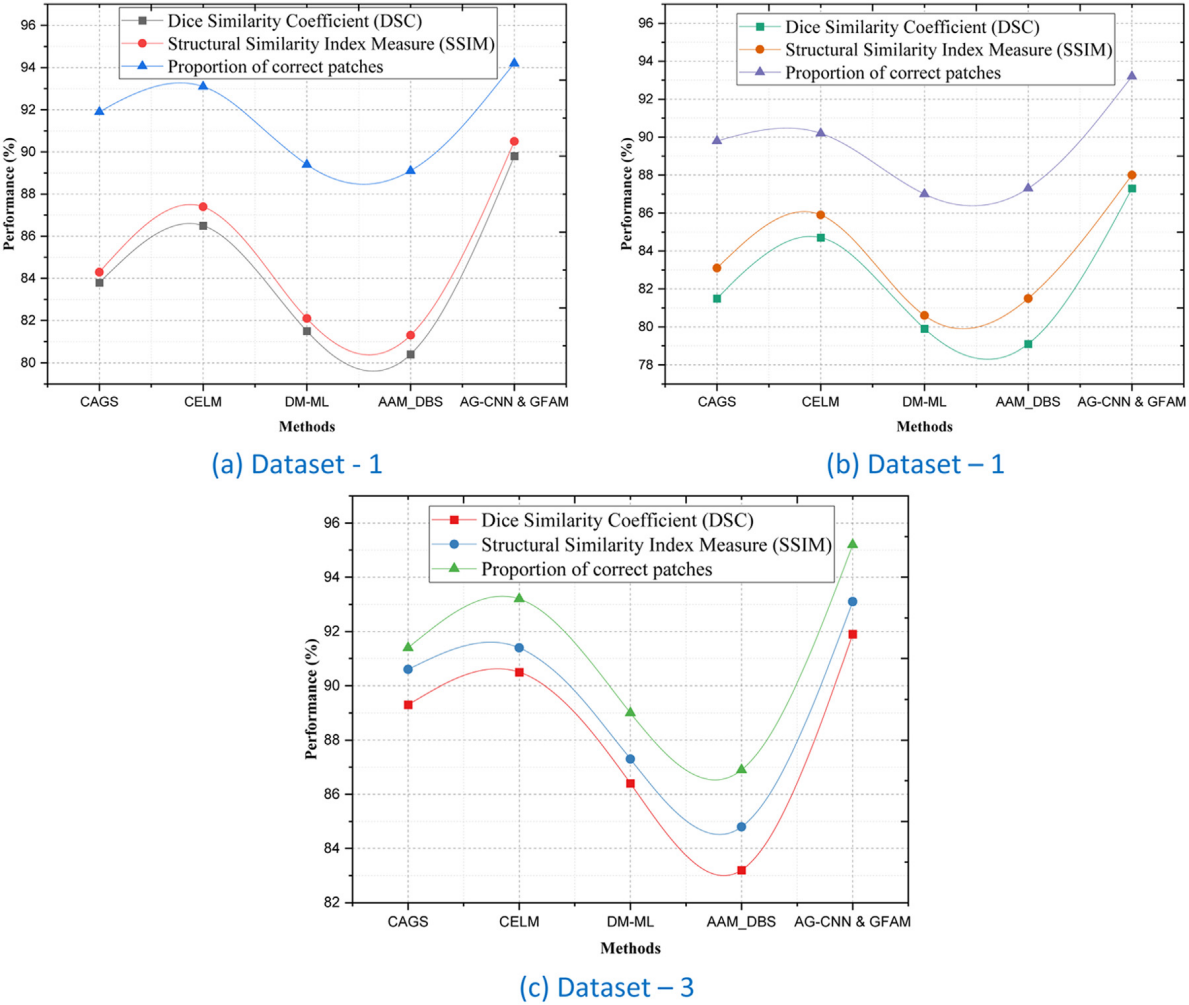
Classification of Segmentation and Structural Accuracy.

To ensure the credibility of the proposed AG-CNN & GFAM framework, three more evaluation criteria namely Dice Similarity Coefficient (DSC), Structural Similarity Index Measure (SSIM), and Proportion of Correct Patches (PCP) were also employed. These metrics as a whole give an account of the segmentation accuracy, structural integrity, and correctness of the predicted liver tumor region in regions. The Dice Similarity Coefficient (DSC) measures the overlap between the predicted masks and the ground truth in terms of the degree of overlap with higher values indicating an ideal fit. For Dataset 1, the proposed learning models AG-CNN & GFAM obtained DSC 89.80% which is even superior to other methods as CELM and DM-ML with 86.50% & 81.50% respectively as shown in Table 2. This is enhancement of 3.3% in CELM and an enhancement of 8.3 % in the model in discriminating the tumor region. Likewise, for Dataset 3, AG-CNN & GFAM recorded a DSC of 91.9 % which was 1.4% better than the CELM (90.5%). The Structural Similarity Index Measure (SSIM) assesses the structural accuracy of the segmentation output more accuracy with paying attention to the texture and spatial changes. As shown in Dataset 1, SSIM was scored by AG-CNN & GFAM at 90.5% which is higher than CELM, with a score of 87.4%, and DM-ML with a score of 82.1% as shown in Fig. 8. Because attention-guided mechanisms and genomic integration, the model demonstrated 3.5% improvement to that of CELM in terms of structural similarity. Finally in dataset 3 while processing images, the combination of AG-CNN & GFAM proposed achieved an SSIM index of 93.1 % thereby confirming its efficacy in preserving the structural impressions of the datasets.

**Table 2:** Classification of Segmentation and Structural Accuracy.

Datasets	Methods	Dice Similarity Coefficient (DSC)	Structural Similarity Index Measure (SSIM)	Proportion of correct patches
Dataset 1	CAGS	83.8	84.3	91.9
	CELM	86.5	87.4	93.1
	DM-ML	81.5	82.1	89.4
	AAM_DBS	80.4	81.3	89.1
	AG-CNN & GFAM	89.8	90.5	94.2
Dataset 2	CAGS	81.5	83.1	89.8
	CELM	84.7	85.9	90.2
	DM-ML	79.9	80.6	87
	AAM_DBS	79.1	81.5	87.3
	AG-CNN & GFAM	87.3	88	93.2
Dataset 3	CAGS	89.3	90.6	91.4
	CELM	90.5	91.4	93.2
	DM-ML	86.4	87.3	89.0
	AAM_DBS	83.2	84.8	86.9
	AG-CNN & GFAM	91.9	93.1	95.2

Proportion of Correct Patches (PCP) quantifies the correct lesion contour delineation at a fine grain level, on an individual patch basis. Similar results are observed for Dataset 1 for which both AG-CNN & GFAM has the highest PCP of 94.2 % which is higher than CELM (93.1%) and DM-ML (89.4 %). This trend is consistent across the datasets, with AG-CNN & GFAM outperforming the other methods: The PCP was 95.2% in Dataset 3, +2% compared to CELM, 93.2%. These results clearly indicate that AG-CNN & GFAM are effective for handling segmentation problems. The use of attention mechanisms in the AG-CNN helps to identify and localize the tumor area optimally, while GFAM provides further information about subtypes of the tumor. Together they lead to consistently higher performance characteristics with significant improvement in segregation and structural integrity. In present datasets, there is a 3-6% enhancement on average by the proposed framework over conventional approaches, which validates its effectiveness for clinics feasible liver tumor anticipation.

## Limitations

As shown in the proposed framework, there is a substantial improvement in liver tumor prediction, though there are some limitations that need further research. Pertaining to the model's dependence on large high-quality datasets comprising of CT imaging and genomic data, it may not be readily applicable to other clinical settings where data is scarce or imaging protocols differ significantly. To address this, future work will focus on developing advanced data augmentation strategies and domain adaptation techniques to improve the model's scalability and adaptability to diverse datasets. Furthermore, despite the fact that both AG-CNN and GFAM provide very accurate and precise results in diagnostics, the computational cost of the proposed framework may be quite a concern in terms of the feasibility of applying the methods to clinical diagnosis in real-time setups in developing countries with inadequate facilities and equipment. Lightweight model architectures will be explored to reduce computational demands while preserving model performance, ensuring broader clinical applicability.

## Conclusion and Future Scope

Here, we proposed a new approach to liver tumor prediction, which consists of the Attention-Guided Convolutional Neural Networks (AG-CNNs) and Genomic Feature Analysis Module (GFAM). Our method overcomes the problem of tumor heterogeneity and multi-modal data by integrating both high-resolution imaging data and molecular information from genomic analysis. The AG-CNN module performs best for the segmentation of tumor regions with a higher level of accuracy and uses attention for spatial as well as morphological understanding. The GFAM module is also able to recognize other molecular markers at the same time, providing essential supportive information for accurate discrimination of liver tumor subtypes and providing useful prognostic information. Based on the results, extensive testing on TCIA, LiTS, and CRLM datasets supported the efficiency of our framework. On all datasets, AG-CNN & GFAM gave better accuracy, F1-Score, and recall up to 10% higher than the competing methods including CELM, CAGS, and DM-ML. Importantly, our model had a Dice Similarity Coefficient of 91.9%, F1-Score 96.2 %, and the model performance was significantly excellent in terms of robustness on various datasets further demonstrating its potential for clinical applications. Apart from enhancing the predictive performances, our proposed integration method enhances complementary information on the tumor's characteristics and can be employed as a tool to support clinical decisions. This framework is a giant leap in the field of precision oncology, as the exact diagnosis, coarse classification of subtypes, as well as future targeted treatment plans for liver tumors could be efficiently and accurately prophesied using this tool. Future work will explore the customization of treatment plans based on the model's prediction results, which would require additional clinical trials and validation studies to assess its direct impact on treatment decisions. This foundation may be further developed in future studies by testing other modalities, as well as implementation in clinical practice more universally in cancer diagnosis and treatment. Future works could be concerned with improving the architecture related to computational complexity and exploring an effective light-level model without performance degradation. Future work will also involve conducting detailed ablation experiments to evaluate the independent contributions of the AG-CNN, GFAM, and attention-guided mechanisms. This will provide deeper insights into the model's architecture and help identify opportunities to further optimize performance.

## Ethics statements

In this Manuscript no, human participants or animals their data or biological material, are not involved.

## CRediT author statement

**S. Edwin Raja:** Methodology, Software, Validation, Field study, **J. Sutha:** Visualization, Writing-Original draft preparation, **P. Elamparithi:** Conceptualization, Data curation, **K. Jaya Deepthi:** Methodology, Writing-Reviewing and Editing, **S D Lalitha:** Writing-Reviewing and Editing, Investigation.

## Declaration of competing interest

The authors declare that they have no known competing financial interests or personal relationships that could have appeared to influence the work reported in this paper.

## Data Availability

No data was used for the research described in the article.
